# Influence of organic plant breeding on the rhizosphere microbiome of common bean (*Phaseolus vulgaris* L.)

**DOI:** 10.3389/fpls.2023.1251919

**Published:** 2023-10-25

**Authors:** Hayley E. Park, Lucas Nebert, Ryan M. King, Posy Busby, James R. Myers

**Affiliations:** ^1^Department of Horticulture, Oregon State University, Corvallis, OR, United States; ^2^National Clonal Germplasm Repository, Agricultural Research Service, United States Department of Agriculture, Corvallis, OR, United States; ^3^Department of Botany and Plant Pathology, Oregon State University, Corvallis, OR, United States

**Keywords:** crop management, microbiome, rhizosphere, breeding history, snap bean

## Abstract

**Introduction:**

We now recognize that plant genotype affects the assembly of its microbiome, which in turn, affects essential plant functions. The production system for crop plants also influences the microbiome composition, and as a result, we would expect to find differences between conventional and organic production systems. Plant genotypes selected in an organic regime may host different microbiome assemblages than those selected in conventional environments. We aimed to address these questions using recombinant inbred populations of snap bean that differed in breeding history.

**Methods:**

Rhizosphere microbiomes of conventional and organic common beans (*Phaseolus vulgaris* L.) were characterized within a long-term organic research site. The fungal and bacterial communities were distinguished using pooled replications of 16S and ITS amplicon sequences, which originated from rhizosphere samples collected between flowering and pod set.

**Results:**

Bacterial communities significantly varied between organic and conventional breeding histories, while fungal communities varied between breeding histories and parentage. Within the organically-bred populations, a higher abundance of a plant-growth-promoting bacteria, *Arthrobacter pokkalii*, was identified. Conventionally-bred beans hosted a higher abundance of nitrogen-fixing bacteria that normally do not form functional nodules with common beans. Fungal communities in the organically derived beans included more arbuscular mycorrhizae, as well as several plant pathogens.

**Discussion:**

The results confirm that the breeding environment of crops can significantly alter the microbiome community composition of progeny. Characterizing changes in microbiome communities and the plant genes instrumental to these changes will provide essential information about how future breeding efforts may pursue microbiome manipulation.

## 1 Introduction

Global efforts to improve the sustainability of crop production include organic agriculture as an element of proposed solutions. Several meta-analyses confirm that organic agriculture requires fewer non-renewable resources (Badgley et al., 2007; Smith et al., 2015), reduces nonpoint source pollution from agrochemicals (Friedman, 2007), provides producers with opportunities for higher profit margins (Shreck et al., 2006), improves soil and on-farm biodiversity (Bengtsson et al., 2005; Puissant et al., 2021), and has the potential to improve conditions for farmworkers and rural communities (Shreck et al., 2006).

Concerns remain regarding the organic yield gap, estimates of which suggest that organic yields produce between 66-91% of conventional yields (Badgley et al., 2007; Seufert et al., 2012; Ponisio et al., 2015; Röös et al., 2018). This range is influenced by numerous factors (i.e. crop type, life cycle, and management practices utilized); however, some clear trends exist. Namely, the amount of plant available nitrogen and the scale of perennial weed pressure are the greatest driving forces behind the yield gap (Kravchenko et al., 2017; Knapp & van der Heijden, 2018). Several approaches are proposed to reduce the yield gap including increasing organic N inputs, maintaining soil pH for optimum nutrient uptake, adoption of precision farming and other technologies in organic systems, and the breeding and development of new cultivars that are well-suited to organic farm conditions (Ponisio et al., 2015; Kravchenko et al., 2017; Knapp & van der Heijden, 2018; Röös et al., 2018).

Many cultivars currently used in organic systems are developed in breeding programs geared toward conventional agriculture and are later moved to the organic market after trialing within target environments (Lammerts van Bueren & Myers, 2012). A growing body of research suggests that direct selection for new cultivars within organic environments may be a more effective way to develop varieties with consistently high performance in organic systems (Murphy et al., 2007; Wolfe et al., 2008; Lammerts van Bueren et al., 2011). Specific traits of interest include amenability to mechanical cultivation, spreading leaf canopies for weed suppression, nutrient-seeking root architecture, and an ability to form a resilient microbiome (Lammerts van Bueren et al., 2011; Baker et al., 2020; Keijzer et al., 2022)

Snap beans, *Phaseolus vulgaris*, are the form of common bean grown for their stringless, low-fiber vegetable pods. These include green beans, wax beans, as well as flat-podded Romano beans. In conventional systems snap beans are coated with fungicide seed treatments and pre-emergent herbicides are applied prior to germination, reducing the need for vigorous seedlings that can outgrow early pest pressure. Synthetic fertilizers applied throughout the snap bean growth cycle reduce the need for roots that expand appropriately to seek out nutrients (Duncan et al., 1960; Lammerts van Bueren et al., 2011). Furthermore, breeding exclusively within conventional systems with high nitrogen availability has resulted in unintentional loss of function of the legume genes responsible for supporting symbiotic relationships with nitrogen-fixing soil bacteria (Kiers et al., 2007).

Within the Pacific Northwest, and western Oregon specifically, snap bean production thrives, as a product of reduced disease pressure and adequate irrigation capabilities. Oregon ranked third nationwide in 2021 for organic snap bean production, and second for production of organic snap beans intended for the processing market (USDA National Agricultural Statistics Service, 2022). Snap bean production in western Oregon is characterized by cool, wet spring conditions that hamper stand establishment and early performance. Factors such as seed color, seed coat thickness, and sprawling plant growth habit may be favorable for production in this climate (Cirak and Myers, 2021); however, there is a scarcity of information regarding the plant-associated microbiome and adaptation to organic production.

Plant-microorganism relationships include a wide breadth of symbioses that are parasitic, mutualistic, or commensal in nature. These relationships, and the community assembly, are complexed by the broader interspecies dynamics within a given microbiome as well as ambient environmental pressures (Busby et al., 2017; Dini-Andreote & Raaijmakers, 2018). A growing interest in harnessing the crop microbiome to bolster agricultural production has led to inquiry into various components of plant-microbiome interactions, including those which take place at the nexus of the plant root and surrounding soil, a region known as the rhizosphere.

The rhizosphere is an influential region for crop performance due to the presence and impact of soilborne pathogens. In common bean, persistent biotic pressures, such as the fungal pathogens *Rhizoctonia solani*, *Pythium* spp., *Fusarium solani*, and *Fusarium oxysporum*, are responsible for poor stand establishment and yield reductions. These pathogens are soilborne, and are generally controlled through fungicidal seed coatings, crop rotations, cultural management, and planting of resistant cultivars (Mohamed et al., 2016; Bartholomäus et al., 2017; Alves Eloy et al., 2021). Not all soil microorganisms are detrimental to host plants, however, and a growing number of beneficial plant-associated species have been identified. Recent research suggests that the microbiome community can be altered via biocontrol inoculation or by breeding plants to support specific taxa in the rhizosphere that are antagonistic towards the pathogenic soilborne microorganisms (Mayo et al., 2015; Mendes et al., 2018a; Mendes et al., 2018b). In the case of *F. solani*, relationships between *P. vulgaris* and arbuscular mycorrhizal fungi (AMF) were tied to pathogen suppression, and this symbiosis appeared to be amenable to increased occurrence through selective plant breeding (Eke et al., 2016; De Vita et al., 2018).

The most notable of symbioses found in legume crops are those held with rhizobia, the atmospheric nitrogen-fixing bacterial species that inhabit legume roots. These microbiota form mutualistic relationships with a wide array of legumes by invading the root tissue, multiplying in modified root compartments known as nodules, and providing the host with plant-available nitrogen (Martínez-Romero, 2003). Relationships with rhizobia are integral to the common bean life cycle, as the microorganisms provide up to 50% of plant nitrogen needs (Rondon et al., 2007). In snap beans this nitrogen provisioning is somewhat reduced, due to a high degree of promiscuous nodulation that leads to the development of few nodules capable of fixing nitrogen (Beshir et al., 2015; Moura et al., 2022).

Common bean cultivars that are available on the market are almost exclusively derived from breeding programs that operate with a conventional, high-input approach to crop management. High-input breeding of legumes appears tied to a relaxation of plant defense mechanisms that historically enforced nodulation with specific rhizobial taxa to provide nitrogen to the host. However, modern legume germplasm nodulates more promiscuously, forming relationships with rhizobia that are inappropriate for the crop species and unable to fix nitrogen for the host (Kiers et al., 2007; Moura et al., 2022). In modern snap bean germplasm, there are several cultivars on the market that are known to be non-modulating altogether, including Pismo and Cosmos.

Breeding for a resilient rhizosphere microbiome may be valuable for any agricultural system, but within the context of organic production this work is critical. While in conventionally managed environments pathogens such as *F. oxysporum*, *F. solani*, and *R. solani* can be managed in part through fungicide applications, organic producers are limited to crop rotations, resistant cultivar selection, and other physical management techniques. Thus, disease resistance conferred by the rhizosphere microbiome is pertinent. While the historic impacts of microevolutions due to selection under high-input management have been demonstrated in both cereal and legume crops, there is an absence of research regarding the impacts of varietal selection under organic management on crop recruitment of a specific rhizosphere microbiome. The objective of this research was to explore whether similar genetic populations with different breeding histories had similar or different microbiome assemblages.

## 2 Materials and methods

### 2.1 Development of recombinant inbred populations

The four populations utilized in this experiment were derived from two initial crosses made in the winter of 2015. The parents used in crosses paired a Modern, Elite Snap Bean To An Older Cultivar With Good Performance In Organic And Low-Input Conditions, As Described In **Supplementary Table 1**. All the parental populations were determinate (bush) beans and produced medium sized pods. The elite parent Hystyle has resistance to Bean Common Mosaic Virus (BCMV), Bacterial Brown Spot, and Curly Top Virus, while the elite parent OR5630 has resistance to BCMV, alone (Cornell University, 2012). The older cultivar Provider is resistant to BCMV, while the older cultivar Black Valentine was recently reported to have some resistance to the root rot complex found in western Oregon (Huster et al., 2021).

The two F_1_ generations were grown at the Vegetable Research Farm in Corvallis, OR in the summer of 2015, and the four research populations were created from 500 seeds of bulked F_2_ seed. Beginning in the summer of 2016 the populations were split into either organic or conventional management and followed a series of generation advances with only natural selection, described in **Supplementary Table 2**.

### 2.2 Study site and experimental design

Snap beans were planted on the Lewis Brown Research Farm at latitude of N44.555881 and longitude of W123.214569 into a Chehalis silty clay loam. This field has been certified organic for over a decade and was previously planted to barley (*Hordeum vulgare*) in 2019. To minimize variation in the seed microbiome, all seeds used for this study were obtained from plants grown in the Oregon State University greenhouses in January of 2020. Treatment in the greenhouse included fertilizer applications of organic and conventional soils and fertilizers, in alignment with the breeding history of the accessions.

A subset of 40 F_7_ snap bean accessions derived from the four research populations along with the four parents were grown. The snap beans were planted on May 21, 2020 and root samples were collected the first week of July, which correlated with flowering and early pod set. The field was managed organically, regardless of the breeding history of the individual accessions. Beans were planted in 3m (10 ft) plots, with 30.5 cm (12 in) spacing between plots and 76 cm (30 in) spacing between rows. Plots were fertilized on June 1, 2020, with Nutri-Rich fertilizer (8-2-4 N-P-K) at a rate of 1,159 kg ha^-1^. Following the fertilizer application, plots were thinned to approximately 15 cm spacing between plants. Mechanical cultivation was used to control weeds throughout the growing season. Plots were irrigated approximately once a week with the application of about 2.5 cm water through solid-set overhead sprinklers.

### 2.3 Soil sampling

Soil samples were collected in April of 2020, prior to fertilizer applications. These samples were sent to the Soil Health Laboratory at Oregon State University and tested for general soil quality and nutrient composition. A second set of soil samples was collected one week prior to root sample collection. These soil samples were tested for general quality (i.e., macronutrient and micronutrient content, organic matter, etc.), and soil microbial assessment.

### 2.4 Rhizosphere soil sample collection and DNA extraction

In July 2020 root samples, along with the associated rhizosphere soil, were collected from the subset of 40 RILs along with the four parents. Two days prior to sample collection the field was irrigated to allow for easier harvest of root material. Samples were collected following a modified harvest protocol designed for the study of perennial grass roots (McPherson et al., 2018). Three plants were randomly selected for harvest from each plot. Each plant, including roots, was collected by digging a soil core with a radius of ~20 cm around the sample plant stem. Loose soil was shaken off in the field. Plants were cut at the hypocotyl and plant tissue from above the hypocotyl was discarded.

Using pruning scissors sterilized in 70% EtOH, 4-6 basal roots were clipped from the main root structure. This included the largest basal root in all samples. Samples from each plot were harvested in succession and each plant constituted a separate replicate from a given plot. The replicates from each plot were considered separate samples, and were analyzed without pooling. After each plot was harvested, the workstation was sterilized using 70% EtOH. The root clippings were placed in 50 mL Falcon tubes containing 35 mL autoclaved phosphate buffer and a surfactant, Tween. Falcon tubes were placed on ice and returned to the lab for further processing.

In the lab, the Falcon tubes were vortexed for 2 minutes to release rhizosphere soil. Using sterilized forceps, the roots were removed from the tubes and blotted dry on paper towels. The remaining rhizosphere soil was vortexed for several seconds to resuspend, before pelletizing via centrifugation. A secondary wash with sterile phosphate buffer was done, before pipetting the suspended soil samples into clean 2 mL microfuge tubes. The samples were centrifuged into pellets again, and the supernatant buffer was removed using a pipette. Soil samples were immediately stored at -25°C, until further processing.

### 2.5 Molecular methods for amplicon sequencing

DNA was extracted from the rhizosphere soil samples using the E.Z.N.A.® Soil DNA Kit (Omega Bio-tek, Inc., Norcross, GA, USA), following the manufacturer’s instructions. DNA samples were quantified at 260/280 nm wavelength on the BioTek Synergy 2 Microplate reader to ensure DNA was within the normal range for absorbance and exceeded a quantity of 2ng/uL. Following quantification, the samples were stored at -20°C until further processing.

The samples were sent to the Center for Quantitative Life Sciences (CQLS) at Oregon State University for amplification and sequencing. We used the Earth Microbiome Project protocols and primers for amplification of both the 16s V4 and ITS regions and library prep (Caporaso et al., 2012; Walters et al., 2016; Ul-Hasan et al., 2019). PCR product size was determined by visualization with the TapeStation HS DNA tape and quantified using the protocol outlined in the Illumina Library qPCR Quantification Guide. PCR product quantity was double-checked using the Qubit fluorometer, and samples that failed to show quantities more than the blank used in the standard curve (0.3051ng uL^-1^) were excluded from sequencing steps. Libraries were normalized to the lowest concentration >1ng uL^-1^, then pooled. Any samples<1ng uL^-1^ were added to the pool without normalization. The libraries were then sequenced at the CQLS on Illumina MiSeq (300bp paired-end). Ultimately this process produced two groups of sequence data, as described in **Supplementary Table 3**.

### 2.6 Bioinformatics and statistical analysis

Raw reads were processed for each group by assessing Phred scores, trimming forward and paired reads, denoising data, and creating contigs. Amplicon sequence variants (ASVs) were then generated by removing chimeras and classifying sequences against a reference training database using the RDP Classifier algorithm within the *DADA2* package for R (Callahan et al., 2016a). *DADA2* was selected for use due to the high specificity and resolution compared to other methods (Prodan et al., 2020). ASVs were classified in January 2022 for both 16s and ITS sequences. We classified 16s sequences using the Silva rRNA database with a bootstrap confidence level of 60, and ITS sequences using the UNITE database with a bootstrap confidence level of 60 (Quast et al., 2012; Nilsson et al., 2019). Phylogenetic trees were created for each group of sample sequences using the *phangorn 2.8.1* package, and were compiled with the other ASV and sample data using the *phyloseq* package (McMurdie & Holmes, 2013; Schliep et al., 2017). Sequences determined to be outliers were removed from the phyloseq object, and sequences that were sourced from parents and positive or negative controls were combined in a separate phyloseq object for use in downstream analyses. Chloroplast and mitochondrial DNA were removed from the sequence data before further analysis.

Prior to community composition analysis, the samples underwent taxonomic filtering using the *phyloseq* package and low prevalence reads were removed. These steps are intended to remove rare or spurious ASVs that may result from contamination or sequencing errors (Callahan et al., 2016b). Although this process may diminish the magnitude of some α-diversity comparisons, significant relationships are retained and the results are likely to be more reproducible and comparable across studies (Cao et al., 2021). To explore community diversity and richness within each sample population, we generated Shannon and Simpson metrics on non-rarefied data. This initial exploration of α-diversity was supplemented with a secondary analysis of similar metrics, classified as Richness and Inverse Simpson, using a bootstrapping method to normalize the data (Berry et al., 2017).

All analyses of β-diversity were completed using the *vegan* package, unless otherwise noted (Oksanen et al., 2012). β-diversity, which encompasses the differentiation between two communities or populations, was explored first via unconstrained and constrained ordinations. For this we utilized a principal correspondence analysis (PCoA) and a canonical analysis of principal coordinates (CAP) to generate the unconstrained and constrained ordinations, respectively, for both breeding history and parentage. ANOVAs were run on the ordinations of each sample type constrained under each set of explanatory variables. Unconstrained ordinations, though valuable in initial pattern assessment, are limited in power by data variability and structure, and while constrained analysis provides a clearer view of group-based variation, it does not capture comprehensive patterns in multivariate data (Anderson and Willis, 2003). Neither approach accounts for the influence of total counts on patterns within the data, thus a secondary analysis of β-diversity was run utilizing centered-log ratio (CLR) transformations. The CLR-transformed data was used to create a Euclidean distance matrix on which permutational analyses of variance (PERMANOVAs) were run to test the significance of various explanatory variables. The homogeneity of variance was tested for each of the explanatory variables using the *betadisper* function.

Following significance tests of explanatory variables, we sought to identify biologically relevant information on the specific taxa responsible for the variation between sample groups. To do this, we utilized the sparse partial least squares-discriminant analysis (sPLS-DA), with a maximum distance method, from the *mixOmics* package (Rohart et al., 2017). The sPLS-DA method is an extension of a PLS regression analysis that includes a LASSO penalization to select variables, which in the context of this study is ASV taxon groups (Lê Cao et al., 2011). It is further extended to become a discriminant analysis by encoding the response matrix with class variables, such as organic and conventional breeding history. The taxon groups most responsible for shifts in centroids can then be extracted and are interpreted to be the taxa most responsible for community composition differences. Following our discriminant analysis, the 10 ASV groups most responsible for shifts between the organic and conventional classes along each principal component were compiled. The raw nucleotide sequence for each ASV group was run through the NCBI BLAST taxonomy search, and additional information generated from the sequence regarding identity and putative function were collected (Schoch et al., 2020).

### 2.7 Heritability

A series of ANOVAs were run to compute variance components for the estimations of narrow-sense heritability. Narrow-sense heritability was estimated for several microbiome metrics, including those representing both α- and β-diversity.

Narrow-sense heritability was estimated for each category of data, using the following formula:


$$h^{2}=\sigma_{G}^{2}/\sigma_{G}^{2}+\sigma^{2}$$

where $\sigma_{G}^{2}$ is the estimated genotypic variance component (M. Singh et al., 1993).

Heritability was calculated for each trait on a family means basis using the *dplyr* and *EnvStats* packages for R (Wickham, 2009; Millard, 2013). An analysis wherein all model components were treated as random effects, was used to obtain the variance components.

## 3 Results

### 3.1 Soil tests

In April of 2020 the soils in the Lewis Brown Farm research plots had a pH of 6.4, along with several other nutrient and organic matter metrics seen in **Supplementary Table 4**. The first test took place one month before planting, and prior to fertilizer and compost application. The second test, in July 2020, indicated a drop in pH to 6.25 along with changes in several other nutrient metrics. Notably, phosphate decreased slightly while potassium increased. Microbial respiration was 35.9 µg CO_2_-C/g dry soil/day. Comparing this to other estimates of microbial respiration, this is considered very low soil activity. Microbial respiration increases with temperature, therefore the value would have increased into August, although may not have exceeded moderately low activity at that point either.

### 3.2 Rhizosphere community composition

The four populations of *Phaseolus vulgaris* hosted a diverse community of fungi and bacteria. After the removal of outliers, controls, and parents, the analysis identified 21 bacterial phyla comprised of 809 unique ASVs and 4 fungal phyla comprised of 129 unique ASVs. Bacterial communities were dominated by Proteobacteria (47% of community), followed by Bacteroidota (18.5% of community), Acidobacteriota (6.3% of community), and Actinobacteriota (6.2% of community). Among fungal communities Basidiomycota dominated (53.5% of community), followed by Ascomycota (34.9% of community) and Mortierellomycota (9.3% of community).

**Figure 1:**
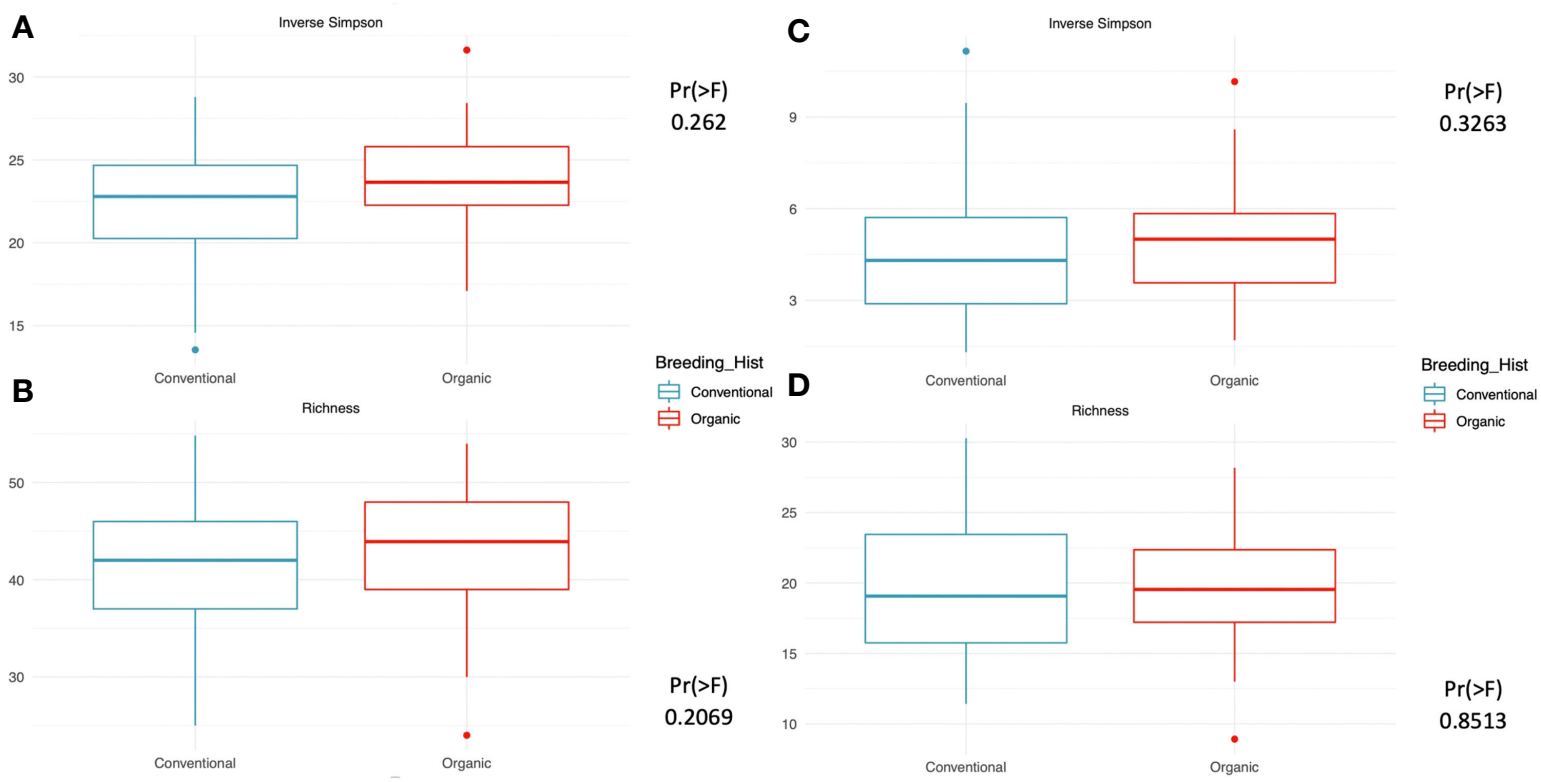
Measures of within-group richness and evenness of 16s and ITS sequences in rhizosphere soil samples from snap beans of conventional and organic breeding histories, collected from Lewis Brown Farm in Corvallis, OR in 2020. The Inverse Simpson metric describes the evenness within groups, which is elevated but not significantly different between the two breeding histories for either the **(A)** 16s sequences or the **(C)** ITS sequences. Richness within groups was slightly higher in the **(B)** 16s sequences and similar for the two breeding histories in the **(D)** ITS sequences.

Our assessment of within-group microbiome diversity (α-diversity) for the two breeding history groups identified trends across both 16s and ITS communities. Broadly, the organically bred populations displayed slightly more evenness in both the micro- and myco- biomes, as seen in **Figures 1A, C**). This trend was also observed in the richness values determined for the 16s sequences, **Figure 1B**), although it was diminished in the ITS sequence analysis, **Figure 1D**). While the increase in evenness and richness was consistent across metrics and sample types, the increases were not statistically significant.

**Figure 2:**
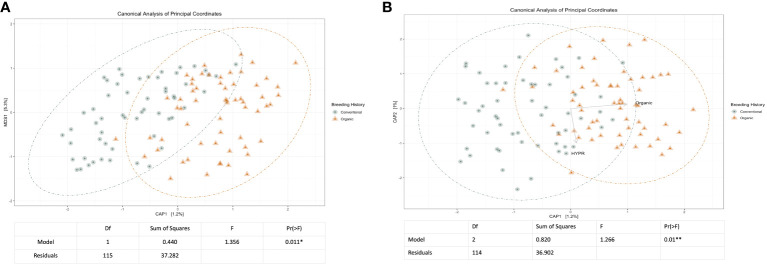
**(A)** Canonical analysis of principal components ordination of snap bean rhizosphere samples based on breeding history, generated from 16s sequences, with ANOVA of the model shown below. **(B)** Canonical analysis of principal components ordination of snap bean rhizosphere samples based on breeding history and cross, generated from 16s sequences, with ANOVA of the model shown below.

**Figure 3:**
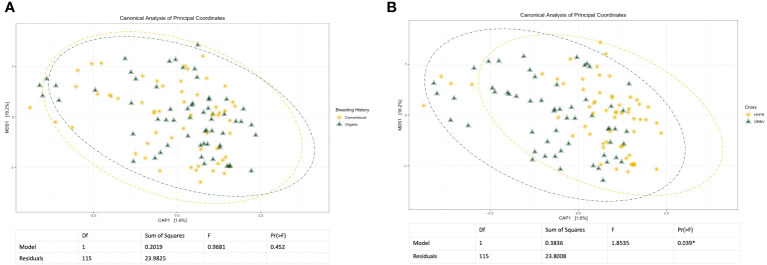
**(A)** Canonical analysis of principal components ordination of snap bean rhizosphere samples based on breeding history, generated from ITS sequences, with ANOVA of the model shown below. **(B)** Canonical analysis of principal components ordination of snap bean rhizosphere samples based on cross, generated from ITS sequences, with ANOVA of the model shown below.

Initial and exploratory unconstrained ordinations were generated using a PCoA. These indicated a general shift in the bacterial microbiome associated with breeding history, as well as a smaller shift associated with cross, as seen in **Supplementary Figures 1**, **2**. Similar shifts were observed in the mycobiome, as seen in **Supplementary Figures 3**, **4**, wherein both breeding history and cross were associated with community shifts. Subsequent constrained analyses accounted for these shifts within the model of the ordination, and were paired with ANOVAs to test the significance of community shifts. For the bacterial microbiome, breeding history retained an observable shift in community composition (**Figure 2A**) and the ANOVA indicated the shift described by breeding history was significant at α< 0.05. When the model included both breeding history and cross, a heightened shift was observed in the ordinations (**Figure 2B**) and the paired ANOVA found the shift to be significant at < 0.01. Although cross amplified the breeding history shift, when analyzed independently it was not found to cause a shift in ordination of any significance. Exploring shifts in the mycobiome, the constrained ordination of the data modeled on breeding history showed a very slight shift that was not significant, as seen in **Figure 3A**. The ordination of the data modeled on cross had a markedly larger shift (**Figure 3B**), which was significant at <0.05.

To account for the skew of count data, the relationships identified via the unconstrained and constrained ordinations were further explored through a permutational analysis of variance performed on centered log transformed data. Within the 16s sequence analysis, breeding history was a significant explanatory variable, while cross was not (**Supplementary Table 5**). Both cross and breeding history had normal dispersion as well. For the ITS sequence analysis, both breeding history and cross were significant explanatory variables for the variation seen across the samples (**Supplementary Table 6**). The homogeneity of dispersion for these variables did not indicate any significant, non-normal dispersion.

**Figure 4:**
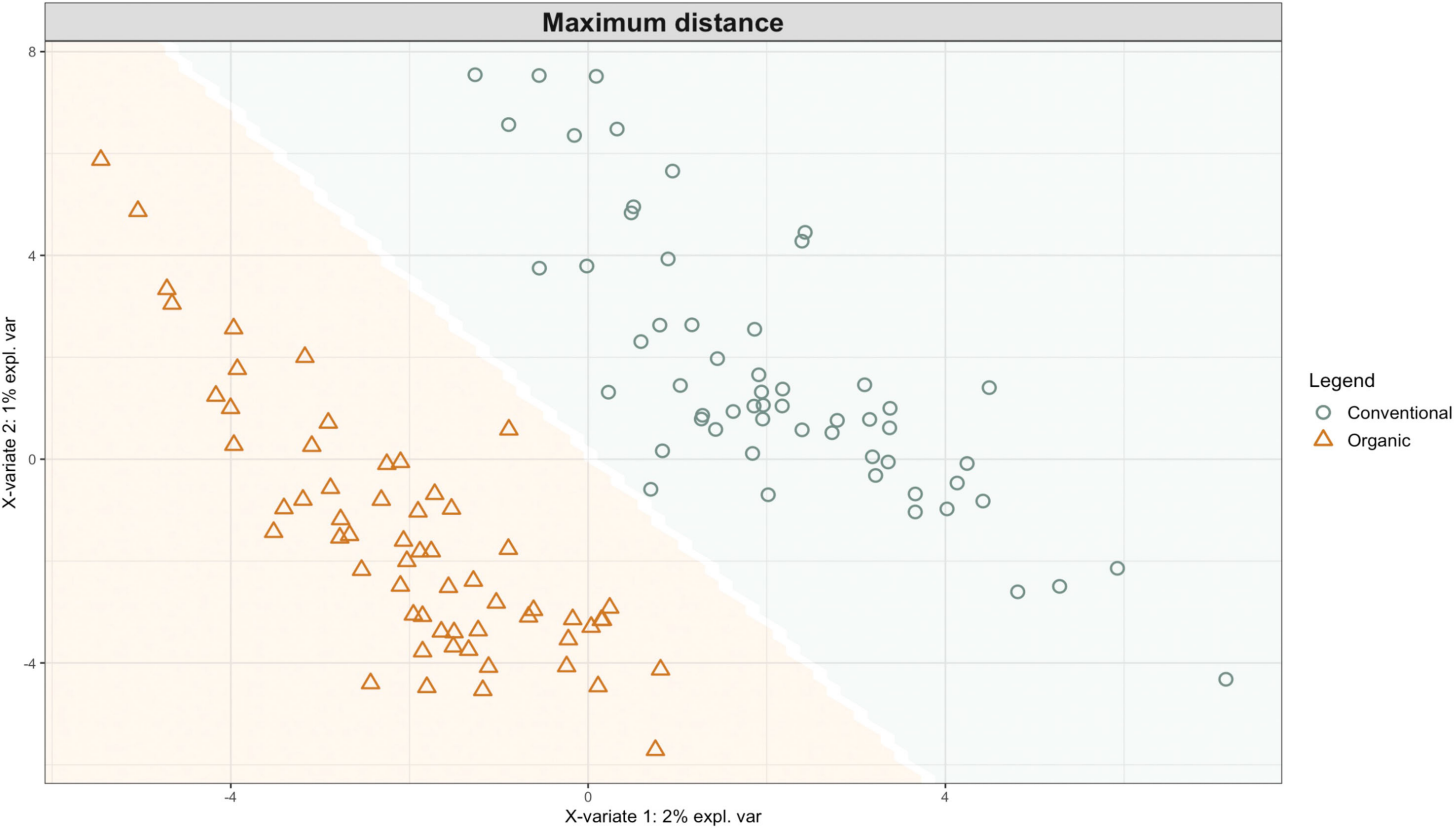
Background prediction areas shown with sample ordinations from snap beans of variable breeding histories, as calculated from maximum distance methods within the sPLS-DA for 16s data of rhizosphere bacteria in organically managed plots at the Lewis Brown Research Farm, OR in 2020.

**Figure 5:**
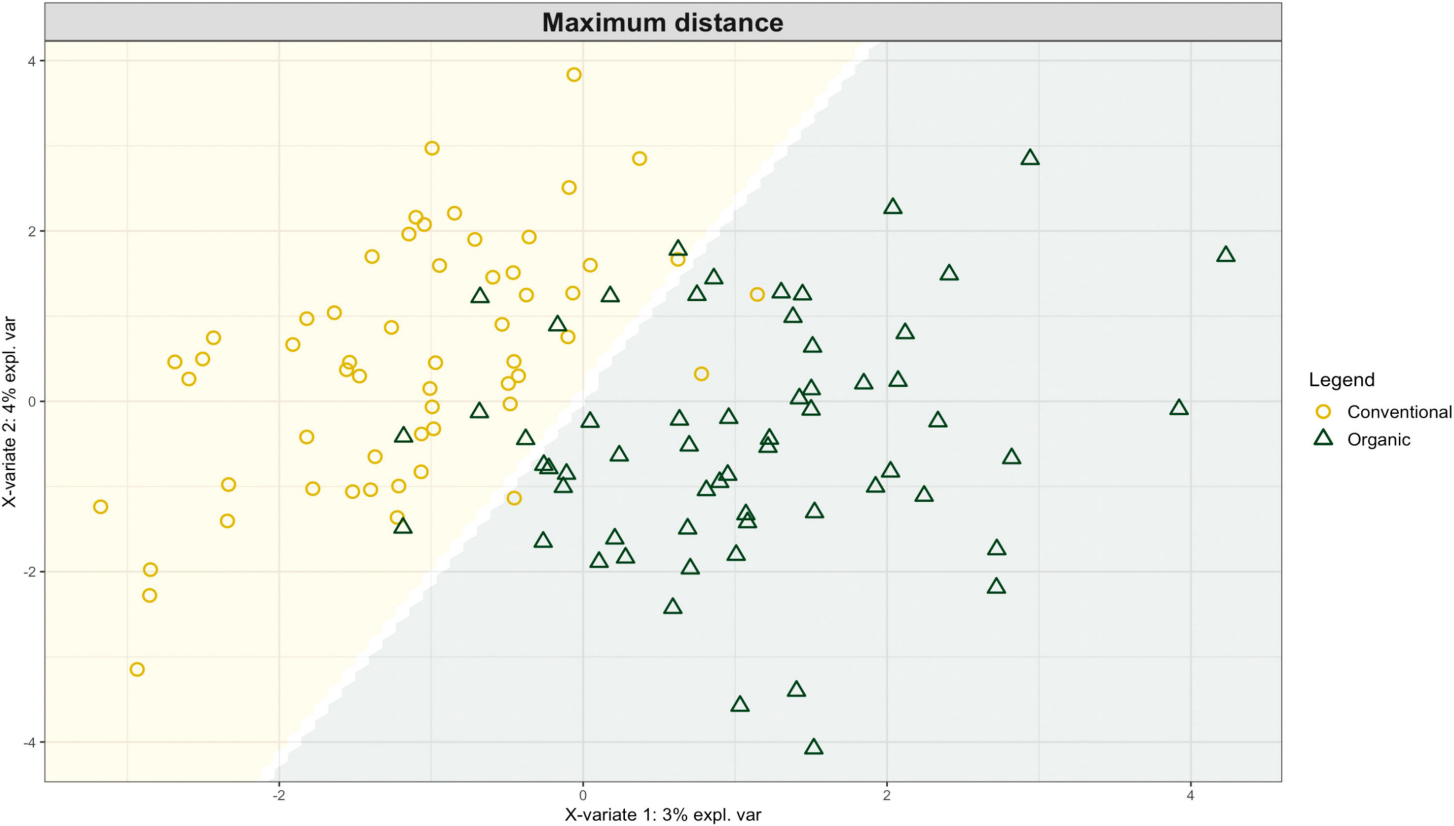
Background prediction areas shown with sample ordinations from snap beans of variable breeding histories, as calculated from maximum distance methods within the sPLS-DA for ITS data of rhizosphere fungi in organically managed plots at the Lewis Brown Research Farm, OR in 2020.

**Figure 6:**
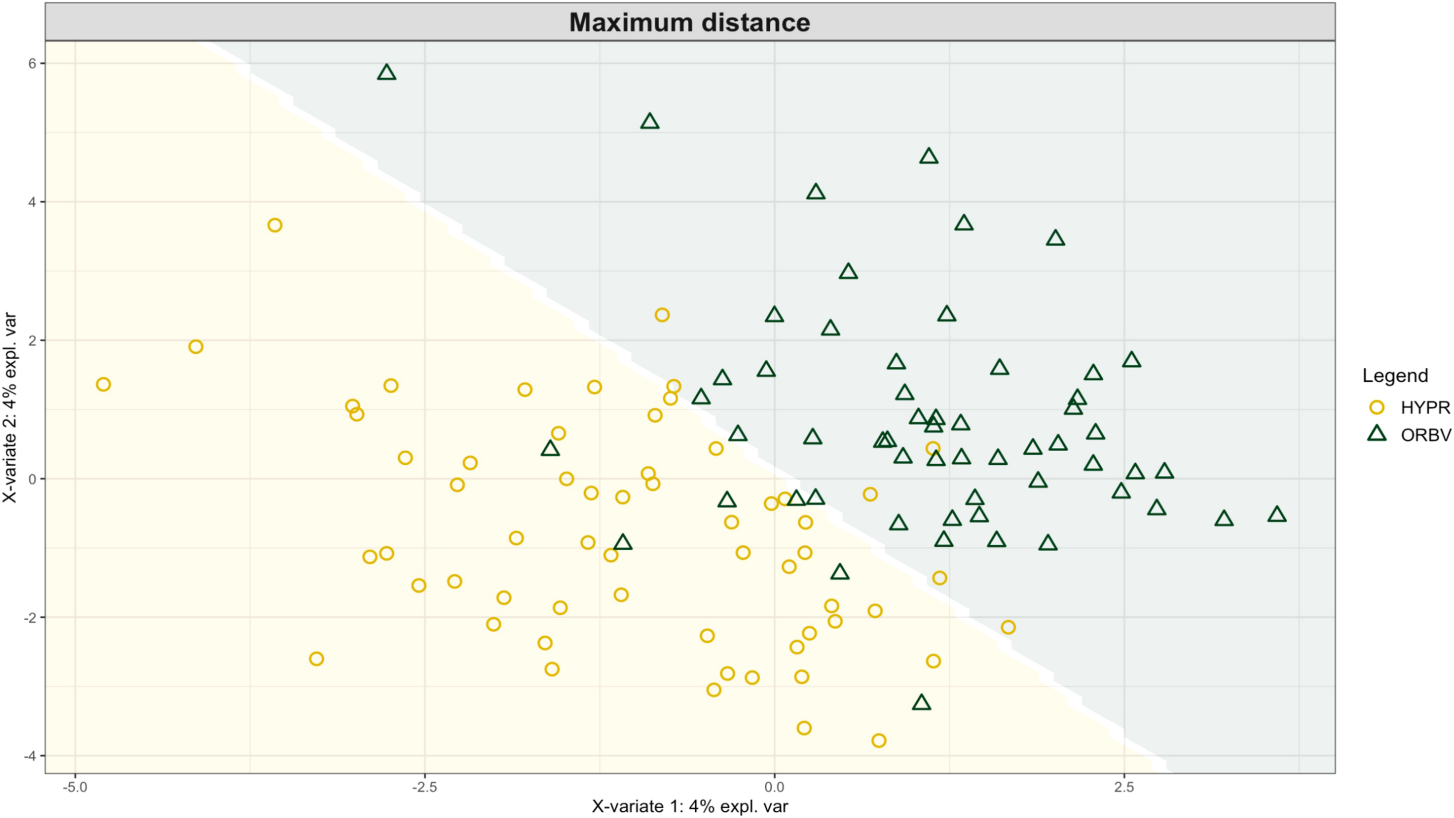
Background prediction areas shown with sample ordinations from snap beans of variable parentage, as calculated from maximum distance methods within the sPLS-DA for ITS data of rhizosphere fungi in organically managed plots at the Lewis Brown Research Farm, OR in 2020.

Exploring these significant relationships further, a sPLS-DA model was tuned to fit the data described in (**Table 1**). Using the classification that was developed through this model tuning, it was possible to estimate the predicted ordination area for each class. For each of the sample types described in **Table 1** a supplemental plot was created that includes the sample ordination along with the predicted background. In **Figure 4** the sample ordination plots cleanly within the predicted background for the 16s sequences with no crossover across the background prediction divide. For the ITS sequences evaluated by breeding history classes, several samples from the organic class were plotted into the conventionally predicted background, as seen in **Figure 5**. The ITS sequences evaluated by parental cross, shown in **Figure 6**, had similar crossover events. In general, for all data sets, most of the samples fell into the ordination space that was predicted using the classification model. The number of crossover events across the predicted background divide in each ordination trends upwards in concert with the error rates for each of the ordination components.

**Table 1:** Model tuning metrics for the sparse partial least squares discriminant analysis (sPLS-DA) used in the identification of differentially abundant fungal and bacterial taxa between snap bean classes, including breeding history and cross.

Sample Type	Class Variables	Taxonomic Level	Distance Method	1st component error rate	2nd component error rate	Number of folds	Number of repeats
16s	Organic, Conventional	Family	Maximum	0.35	0.38	5	50
ITS	Organic, Conventional	Genus	Maximum	0.38	0.37	5	50
ITS	HYPR, ORBV	Genus	Maximum	0.42	0.41	5	50

Model distance method, error rates for the identification of taxa, and model repetitions are reported.

From the ordination loadings it was possible to extract the ASV information that were most responsible for shifts between each of the two classes in each data set. The nucleotide sequence information associated with these loadings was compared to available GenBank sequences using NCBI BLAST. The top match identified in GenBank is reported in **Tables 2**–**4** along with relevant taxonomic and functional information for each sequence.

**Table 2:** Top 10 taxa responsible for variation in principle components axes 1 and 2 of ordinated sample data for 16s sequences, as identified by sPLS-DA of breeding histories, with supplementary GenBank information and possible function in the soil associated with the roots of snap beans grown in organically managed plots at the Lewis Brown Research Farm in 2020.

Ordination Axis	ASV Name	Family Name	Genbank Blast Closest Match	Pathogenicity/ Beneficiality	Possible Function	Increased in	Reference
16s PC1	ASV260	Rhodanobacteraceae	*Tahibacter aquaticus*			Conventional	
16s PC1	ASV58	Comamondaceae	*Albitalea terrae*			Conventional	
16s PC1	ASV221	Rhodanobacteraceae	*Tahibacter aquaticus*			Conventional	
16s PC1	ASV456	Caulobacteraceae	*Caulobacter hibisci*	+	Plant-growth promoting bacteria	Conventional	(Berrios, 2022)
16s PC1	ASV676	Vermiphilaceae	*Legionella nagasakiensis*			Conventional	
16s PC1	ASV551	Micrococcaceae	*Arthrobacter pokkalii*	+	Plant-growth promoting bacteria	Organic	(Krishnan et al., 2016)
16s PC1	ASV365	Comamondaceae	*Zhizhongheella caldifontis*			Conventional	
16s PC1	ASV387	Spirosomaceae	*Emticicia ginsengisoli*			Conventional	
16s PC1	ASV311	Comamondaceae	*Piscinibacter aquaticus*			Organic	
16s PC1	ASV83	Rhodanobacteraceae	*Tahibacter aquaticus*			Conventional	
16s PC2	ASV84	Micrococcaceae	*Arthrobacter pokkalii*	+	Plant-growth promoting bacteria	Conventional	(Krishnan et al., 2016)
16s PC2	ASV459	Rhizobiaceae	*Rhizobium mesoamericanum*	+	Nitrogen-fixing bacteria	Conventional	(López-López et al., 2012)
16s PC2	ASV525	Chitinophagaceae	*Terrimonas rubra*			Conventional	
16s PC2	ASV210	Comamondaceae	*Rhizobacter fulvus*			Conventional	
16s PC2	ASV13	Micrococcaceae	*Arthrobacter pokkalii*	+	Plant-growth promoting bacteria	Organic	(Krishnan et al., 2016)
16s PC2	ASV24	Pedosphaeraceae	*Limisphaera ngatamarikiensis*			Organic	
16s PC2	ASV496	Mycobacteriaceae	*Mycolicibacterium aichiense*			Conventional	
16s PC2	ASV762	Reyranellaceae	*Azospirillum canadense*	+	Nitrogen-fixing bacteria	Conventional	(Mehnaz et al., 2007)
16s PC2	ASV792	Anaerolineaceae	*Bellilinea caldifistulae*			Conventional	
16s PC2	ASV319	Comamondaceae	*Comamonas humi*			Conventional	

**Table 3:** Top 10 taxa responsible for variation in principle components axes PC1 and PC2 of ordinated sample data for ITS sequences, as identified by sPLS-DA of breeding histories, with supplementary GenBank information and possible function in the soil associated with the roots of snap beans grown in organically managed plots at the Lewis Brown Research Farm in 2020.

Ordination Axis	ASV Name	Family Name	Genbank Blast Closest Match	Pathogenicity/ Beneficiality	Possible Function	Increased in	Reference
ITS PC1	ASV98	Laccaria	*Laccaria moshuijun*	+	Ectomycorrhizal	Organic	(Vincenot et al., 2017)
					basidiomycete		
ITS PC1	ASV55	Paraphoma	*Paraphoma ledniceana*			Organic	
ITS PC1	ASV25	Conocybe	*Crepidotus trichocraspedotus*			Organic	
ITS PC1	ASV71	Panaeolus				Conventional	
ITS PC1	ASV7	Rhizoctonia	*Rhizoctonia carotae*	–	Fungal root pathogen	Conventional	(Kurt et al., 2005)
ITS PC1	ASV22	Exophiala	*Exophiala tremulae*			Organic	
ITS PC1	ASV64	Geminibasidium	*Geminibasidium hirsutum*			Organic	
ITS PC1	ASV94	Cadophora	*Cadophora gamsii*	+/-	Plant growth promoter or pathogen	Organic	(Maciá-Vicente et al., 2020)
ITS PC1	ASV33	Laetisaria	*Laetisaria arvalis*	+	Biocontrol of *R. solani*	Conventional	(Lewis & Papavizas, 1992)
ITS PC1	ASV14	Conocybe	*Conocybe moseri*			Organic	
ITS PC2	ASV63	Stephanospora	*Stephanospora xibalba*			Conventional	
ITS PC2	ASV77	Pyxidiophora	*Pleurocatena brevior*	+	Mycoparasite	Organic	(Gams, 2007)
ITS PC2	ASV109	Hormonema	*Hormonema viticola*	+/-		Organic	(Soto et al., 2019)
					Plant growth promoter or pathogen		
ITS PC2	ASV44	Ceratobasidium	*Ceratobasidium ramicola*	–	Plant pathogen	Organic	(Samuels et al., 2012)
ITS PC2	ASV22	Exophiala	*Exophiala tremulae*			Organic	
ITS PC2	ASV65	Byssonectria	*Chaetothiersia eguttulata*			Organic	
ITS PC2	ASV59	Psathyrella	*Psathyrella scanica*			Organic	
ITS PC2	ASV96	Dominikia	*Rhizophagus prolifer*	+	Arbuscular mycorrhizal fungi	Organic	(Pandit et al., 2022)
ITS PC2	ASV87	Ceratobasidium	*Ceratobasidium ramicola*	–	Plant pathogen	Organic	(Samuels et al., 2012)
ITS PC2	ASV8	Minimedusa	*Minimedusa polyspora*	+	Anti-biotic properties	Conventional	(Beale and Pitt, 1990)

**Table 4:** Top 10 taxa responsible for variation in principle components axes PC1 and PC2 of ordinated sample data for ITS sequences, as identified by sPLS-DA of parentage/cross, with supplementary GenBank information and possible function in the soil associated with the roots of snap beans grown in organically managed plots at the Lewis Brown Research Farm in 2020.

Ordination Axis	ASV Name	Family Name	Genbank Blast Closest Match	Pathogenicity/ Beneficiality	Possible Function	Increased in	Reference
ITS PC1	ASV11	Minimedusa	*Minimedusa polyspora*	+	Anti-biotic properties	HYPR	(Beale and Pitt, 1990)
ITS PC1	ASV7	Rhizoctonia	*Ceratobasidium ramicola*	–	Plant pathogen	ORBV	(Samuels et al., 2012)
ITS PC1	ASV104	Phaeosphaeria	*Phaeosphaeria gahniae*	–	Plant pathogen (cereal crops)	ORBV	(El-Demerdash, 2018)
ITS PC1	ASV103	Psathyrella	*Psathyrella undulatipes*			ORBV	
ITS PC1	ASV73	N/A	*Ceratobasidium ramicola*	–	Plant pathogen	ORBV	(Samuels et al., 2012)
ITS PC1	ASV46	Paraphaeosphaeria	*Paraphaeosphaeria sardoa*			ORBV	
ITS PC1	ASV100	Entoloma	*Entoloma kruticianum*			ORBV	
ITS PC1	ASV10	Paurocotylis	*Geopyxis aleurioides*			HYPR	
ITS PC1	ASV82	Paurocotylis				ORBV	
ITS PC1	ASV58	Articulospora	*Calycina alstrupii*			ORBV	
ITS PC2	ASV8	Minimedusa	*Minimedusa polyspora*	+	Anti-biotic properties	HYPR	(Beale and Pitt, 1990)
ITS PC2	ASV63	N/A	*Stephanospora xibalba*			ORBV	
ITS PC2	ASV68	Cheilymenia	*Chaetothiersia eguttulata*			ORBV	
ITS PC2	ASV2	Mortierella	*Mortierella hypsicladia*			HYPR	
ITS PC2	ASV79	Helgardia	*Helgardiomyces anguioides*	–	Plant pathogen (cereal crops)	ORBV	(Crous et al., 2021)
ITS PC2	ASV5	Conocybe				HYPR	
ITS PC2	ASV105	Conocybe	*Parasola crataegi*			ORBV	
ITS PC2	ASV75	Psathyrella	*Psathyrella stercoraria*			ORBV	
ITS PC2	ASV71	Panaeolus				ORBV	
ITS PC2	ASV90	Clarireedia	*Clarireedia bennettii*	–	Plant pathogen (turfgrass)	ORBV	(Salgado-Salazar et al., 2018)

Within the context of the 16s sequences (**Table 2**), there were notable increases in several potentially beneficial taxa in samples derived from beans with both an organic and conventional breeding history. Within conventionally bred accessions, plant-growth promoting bacteria *Caulobacter hibisci* and *Arthrobacter pokkalii* were increased along with the nitrogen-fixing bacteria *Rhizobium mesoamericanum* and *Azospirillum canadense*. The plant-growth promoter *Arthrobacter pokkalii* was increased in multiple instances in the organically bred accessions.

In the context of ITS sequences, both beneficial and pathogenic fungal taxa were present in samples from both breeding histories, as seen in **Table 3**. Among the conventionally bred samples there was an increase in the plant pathogen *Rhizoctonia carotae*, however there was also an increase in the potentially beneficial fungi *Minimedusa polyspora* and *Laetisaria arvalis*, the latter of which is known to have biocontrol properties over *Rhizoctonia*. In the organic bean samples, there were multiple fungi with increased abundance that are known to have beneficial properties in plant-association. Among these are: *Laccaria moshuijun*, a known ectomycorrhizal basidiomycete; *Pleurocatena brevior*, a mycoparasite; and *Rhizophagus prolifer*, an arbuscular mycorrhizal fungus. Additional species were identified in the organically bred samples that had less definitive roles in the rhizosphere mycobiome, such as *Cadophora gamsii* and *Hormonema viticola*. Increases in the plant pathogen *Ceratobasidium ramicola* were also observed.

Shifts in the mycobiome due to parentage or cross are presented in **Table 4**. Among accessions derived from the HYPR cross, the only fungal species that increased was *Minimedusa polyspora*, which is notable for its antibiotic properties. In accessions from the ORBV cross several plant pathogens were elevated: *Ceratobasidium ramicola*, a relative of *Rhizoctonia*; *Phaeosphaeria gahniae* and *Helgardiomyces anguioides*, both pathogens in cereal crops; and *Clarireedia bennettii*, a turfgrass pathogen. There were no notable increases observed among the ORBV accessions in taxa that have putatively beneficial function in the mycobiome.

### 3.3 Heritability

In **Table 5** the narrow-sense heritability estimates for microbiome community composition traits are summarized. β-diversity represents the amount of diversity between sample groups, such as between two breeding histories, and is represented by two principal components derived from an ordination of the trait. Heritability for β-diversity in the 16s data exceeds the estimates made in the ITS data, although this distinction diminishes in the second PC metric used as a trait. The two metrics evaluated for α-diversity, which considers the within sample group diversity, were the taxon richness and the evenness of taxon abundance. Regarding α-diversity, the 16s and ITS samples for each population were quite variable, ranging from low to moderately high heritability. Species richness appeared to have more stable heritability in the fungal environment.

**Table 5:** Narrow-sense heritability estimates for α-diversity and β-diversity metrics, generated from 16S and ITS sequence analysis on snap bean rhizosphere samples.

Population	Sequence Type	PC1 - β-diversity^w^	PC2 - β-diversity^x^	Richness^y^	Evenness^z^
ORBV-O	16s	0.66 ± 0.15	0.76 ± 0.11	0.61 ± 0.16	0.60 ± 0.16
ORBV-C	16s	0.36 ± 0.22	0.93 ± 0.04	0.14 ± 0.22	0.14 ± 0.22
HYPR-O	16s	0.56 ± 0.17	0.57 ± 0.17	-0.01 ± 0.19	-0.01 ± 0.19
HYPR-C	16s	0.56 ± 0.18	0.66 ± 0.15	0.63 ± 0.16	0.63 ± 0.16
ORBV-O	ITS	0.23 ± 0.21	0.65 ± 0.15	0.29 ± 0.21	0.15 ± 0.21
ORBV-C	ITS	0.21 ± 0.22	0.51 ± 0.20	0.38 ± 0.22	-0.08 ± 0.18
HYPR-O	ITS	-0.16 ± 0.15	0.49 ± 0.19	0.32 ± 0.21	0.51 ± 0.19
HYPR-C	ITS	-0.02 ± 0.18	0.59 ± 0.17	0.36 ± 0.21	0.24 ± 0.21

w PC1 describes the movement along the first axis of ordination of centered log transformed sample data.

x PC2 describes the movement along the second axis of ordination centered log transformed sample data.

y Richness describes the quantity of unique taxa in the microbiome.

z Evenness describes the nature of the distribution of taxonomic abundance within samples of the population of interest.

## 4 Discussion

Studies of the microbiome of soils under organic and conventional management reveal consistent trends towards high microbial diversity in organically managed soils (Wang et al., 2016; Sanchez-Barrios et al., 2017). Although there is a growing body of evidence supporting the merits of breeding crops for organic production within organically managed systems, very little work has explored how the breeding environment may impact future microbiome community recruitment of organically bred crops (Osman et al., 2008; Lammerts van Bueren and Myers, 2012; Sanchez-Barrios et al., 2017).

Within the limited available literature, two studies identified significant differences in the rhizosphere microbiome of grain crops that were bred under low-nitrogen management or that were found to have preferable performance in organic conditions. Researchers determined that these rhizosphere differences were a product of increased host-selection of Bacteroidetes in the microbial communities (Visioli et al., 2020; Favela et al., 2021). Our work sought to understand the role of genotypic variation attributable to an organic or conventional breeding environment within *Phaseolus vulgaris*, a crop in which microbiome shifts along the domestication gradient are known to have reduced the presence of Bacteroidetes in favor of Actinobacteria and Proteobacteria (Pérez-Jaramillo et al., 2017). Similar studies in *P. vulgaris* also identified a genotypic role in the formation of the mycobiome (da Silva et al., 2020; Pozo et al., 2021).

Our findings support the hypothesis of differential selection due to breeding under conventional versus organic management. The 16s rRNA sequence analysis of rhizosphere soil samples provided evidence of insignificant increases in within-group microbiome diversity tied to breeding history. Comparing between-group diversity, we found significant shifts in the microbial community within samples from either organic or conventional breeding histories. Of the major species tied to these shifts, several plant-growth-promoting taxa were identified. Sequence analysis of the ITS gene from the same rhizosphere soil samples indicated similar or reduced trends in α-diversity. Changes in the mycobiome between groups revealed the communities significantly shifted along the gradients of both breeding history and parentage. Putatively pathogenic and beneficial fungi were identified in the rhizosphere communities of both organically and conventionally bred beans.

Comparing the communities in terms of parentage provided a clear trend towards more identifiable pathogenic species among the ORBV populations, created from the parents OR5630 and Black Valentine, compared to the HYPR populations. Given the inherent disease resistance of OR5630, and other studies which used Black Valentine as a disease-susceptible control, it is possible that some of this shift could be attributed to this less resilient parent (Scott and Fulton, 1978; Pflieger et al., 2014). However, previous research into the root rot susceptibility of the four parental lines used in the production of the RIL population determined that Black Valentine has high resistance to the specific root rot complex prevalent in western Oregon (Huster et al., 2021).

Disease resistance creates interesting, although somewhat inconsistent, implications in targeted breeding for organic agriculture. In our work, there are notable differences between the disease resistance packages of the two processing bean parents, Hystyle and OR5630, that may influence the micro- and myco- biomes. While both have resistance to Bean Common Mosaic Virus (BCMV), Hystyle has additional reported resistances to Bacterial Brown Spot, and Curly Top Virus (Cornell University, 2012). The influence of these additional disease resistances may help to explain the reduced abundance of pathogenic taxa within the HYPR population samples; however, the known resiliency of Black Valentine against the western Oregon root rot complex suggests that resistance breeding alone cannot explain differences in the microbiome that we observed across populations. Similar inconsistencies have been observed in maize, wherein QTL associated with disease resistance shifted relative abundances of bacteria and fungi but not in a manner that was consistent across time and environments (Wagner et al., 2020). The design of the current study cannot determine the influence of disease resistance on the rhizosphere microbiome, but the differences between the ORBV and HYPR populations may point to fruitful avenues for future research.

As plant breeders seek to identify and utilize rhizosphere-mediated resiliencies, the selection of appropriate host genotypes is essential. In addition to elite germplasm with specific disease resistance packages, wild crop relatives can serve as useful sources for genotypic variation in microbiome recruitment. One study in tomato utilized a population of wild-type tomato inbreds to find specific QTLs associated with microbiome-mediated disease resistance (Pérez-Jaramillo et al., 2016). An assessment of microbial diversity in other *Phaseolus* species, such as *Phaseolus acutifolius* or *Phaseolus filiformis*, may also be useful in identifying QTL associated with drought-tolerance or disease resistance conferred by specific microbial community compositions (Buhrow, 1983; Traub et al., 2017).

The use of RIL populations in this study made it possible to evaluate the changes in α-diversity and β-diversity for heritability. Our findings indicate low to moderate heritability for each of the rhizosphere phenotypes assessed, which builds upon similar findings in the heritability of the phyllosphere microbiome in maize (Wagner et al., 2020). In that work, researchers attributed 4-8% of microbiome variation in the phyllosphere to host genotype, although heritability values for rhizosphere characteristics could not be determined. Given our small sample size (10 samples per population), our heritability results must be interpreted with caution. While our model of replication meets suggested standards, the number of families utilized for the microbiome study are approximately 25% of the number previously shown to be sufficient for precise estimation of heritability (Xie and Mosjidis, 1997).

Due to the complexity of the rhizosphere microbiome, the cost of amplicon sequencing, and the difficulties in reliably estimating heritability, it may be preferable to identify other morphological or physiological host plant phenotypes that can be utilized in indirect selection of the community composition. Specific morphological phenotypes, including root length, quantity of fine roots, and root thickness are associated with changes in the richness and evenness of microbiome composition (Saleem et al., 2016; Pérez-Jaramillo et al., 2017; Saleem et al., 2018). Selection on these traits within target growth environments may prove more productive than attempting to harness plastic microbiome phenotypes. Additionally, understanding the major morphological drivers of the microbiome may point to key management strategies that support the development of a favorable root architecture (Saleem et al., 2018).

Future amplicon sequencing work to investigate the endophytic bacteria and fungi may facilitate a better understanding of the specific microbial taxa recruited by *Phaseolus vulgaris*, particularly in the context of differential breeding history. Endophytes within the seed may deepen our understanding of the early season vigor and resiliency seen in the organically bred bean accessions, as seed endophytes have been shown to alter the seedling microbiome (Verma et al., 2019). Understanding the community of root endophytes may help to determine which taxa are most valuable to plant nutrient acquisition, but also which are most capable of pathogenic invasion of the root tissue.

Our investigation into the four RIL populations identified strong, consistent evidence to support the hypothesis that differential selection under organic and conventional management is responsible for producing micro-evolutions in the snap bean genome related to rhizosphere microbiome characteristics. As the organic industry grows, and requirements for the use of certified organic seed become more stringent, the development of resilient, system-appropriate cultivars is essential. This research and the RIL populations we developed lay the groundwork for future studies to identify specific QTL of interest that can be deployed in organic breeding programs. Beyond such concrete applications, our work is at the forefront of explorations into how the breeding environment alters the future microbiome recruitment capabilities of crops. Irrespective of management approach, understanding how domestication and historic breeding have altered such recruitment capabilities is relevant to future work to develop crop cultivars that are resilient against a host of biotic and abiotic pressures.

## Data availability statement

The datasets presented in this study can be found in online repositories. The names of the repository/repositories and accession number(s) can be found below: https://www.ncbi.nlm.nih.gov/, PRJNA988238 https://www.ncbi.nlm.nih.gov/, PRJNA989655.

## Author contributions

JM and HP conceived of the research. JM, RK, and HP developed research populations. HP carried out the field experiment, sample collection, and DNA extractions. HP did the bioinformatics and statistical analyses with advising and input from LN, PB, and JM. HP wrote the article, with contributions from RK and JM. All authors contributed to the article and approved the submitted version.
