# Supplementary material for: Influence of organic plant breeding on the rhizosphere microbiome of common bean (Phaseolus vulgaris L.)
Source: Front Plant Sci. 2023 Oct 25;14:1251919. doi: 10.3389/fpls.2023.1251919 (PMC10634438; doi:10.3389/fpls.2023.1251919)
Supplement: Supplementary file 1 [file DataSheet_1.docx]

**Supplementary Table 1.** Four snap bean populations created following the F_1_ generation of two snap bean crosses, providing a means of comparing the role of breeding environment during inbreeding.

| Population | Cross | Selection System |
| --- | --- | --- |
| HYPR-O | Hystyle x Provider | Organic |
| HYPR-C | Hystyle x Provider | Conventional |
| ORBV-O | OR5630 x Black Valentine | Organic |
| ORBV-C | OR5630 x Black Valentine | Conventional |

**Supplementary Table 2:** Timeline, generation, location, and project methods for snap bean populations 2015-2021 (Modified from King, 2019).

| Year | Generation | Location^z^ | Systems^x^ | Generation Advance Method |
| --- | --- | --- | --- | --- |
| 2015 | F_1_ | VF | C | Bulk |
| 2016 | F_2_ | LBF | O & C | Single seed descent |
| 2017 | F_3_ | GH & CA | (O & C); C only in CA | Single pod descent |
| 2017 | F_4_ | LBF | O & C | Single plant selection |
| 2018 | F_5_ | GH & CA | C | Bulk |
| 2018 | F_6_ | LBF | O & C | Bulk |
| 2020 | F_7_ | VF | O & C | Bulk |
| 2021 | F_8_ | VF | O & C | Bulk |

^z^ VF = OSU Vegetable Research Farm, LBF = Lewis Brown Research Farm, GH = OSU greenhouses, CA = winter nursery near Indio, CA; ^x^ C=conventional systems, O = organic systems

**Supplementary Table 3:** Groups of raw read data generated from MiSeq sequencing for microbial community composition analysis of the snap bean rhizosphere soil, as collected from bean roots at the Lewis Brown Farm in Corvallis, OR in 2020.

| Group Name | Target Sequence | Sample Source | Number of Samples Sequenced |
| --- | --- | --- | --- |
| 16s Soil | 16s | Rhizosphere soil | 132 |
| ITS Soil | ITS | Rhizosphere soil | 132 |

**Supplementary Table 4:** Soil test results from samples taken from field containing research plots at the Lewis Brown Farm in Corvallis, OR in April and July 2020, including microbial respiration estimates.

|  |  | April 2020^z^ | July 2020^y^ |
| --- | --- | --- | --- |
| Parameter | Units | Organic | Organic |
| C | % | - | 2.1 |
| N | % | - | 0.19 |
| N | kg/ha | 100.88 | - |
| Organic Matter | % | 3.98 | - |
| NO3-N | ppm | - | 39 |
| PO4-P | ppm | 37 | 27 |
| K | ppm | 275 | 436 |
| Ca | ppm | 2732 | 3235 |
| Mg | ppm | 723 | 790 |
| pH | pH units | 6.4 | 6.25 |
| Microbial respiration | µg CO2-C/g dry soil/day | - | 35.9 |

^z^Samples collected in April 2020 were tested by Brookside Laboratories, Inc.in New Bremen, OH; ^y^Samples collected in July 2020 were tested by the Central Analytics Laboratory at Oregon State University in Corvallis, OR.

**Supplementary Table 5.** Permutational analysis of variance (PERMANOVA) and homogeneity of dispersion tests of independent variables influencing the distance matrix of centered-log transformed data, for 16s sequence analysis of rhizosphere bacteria associated with the roots of four snap bean populations grown in organically managed plots at the Lewis Brown Research Farm, OR in 2020.

| **Test** | **Model** | **Effects of Terms** | **Permutations** | **Number of Permutations** |  |  | |  |
| --- | --- | --- | --- | --- | --- | --- | --- | --- |
| PERMANOVA | 16s_clr_dist_matrix^z^ ~ Breeding History + Cross | Marginal | Free | 999 | |  |  |  |
|  | **Independent Variable** | **Degrees of Freedom** | **Sum of Squares** | **R^2^** | | **F** | **Pr(>F)** | **Significance** |
|  | Breeding History | 1 | 1391 | 0.010 | |  | 0.005 | ** |
|  | Cross | 1 | 1244 | 0.009 | |  | 0.111 |  |
|  | Residual | 114 | 130311 | 0.980 | |  |  |  |
|  | Total | 116 | 132946 | 1.000 | |  |  |  |
|  | **Independent Variable** | **Df** | **Sum Sq** | **Mean Sq** | | **F** | **N.Perm^y^** | **Pr(>F)** |
| Homogeneity of Dispersion | Breeding History | 1 | 0.536 | 0.536 | |  | 999 | 0.618 |
|  | Residuals | 115 | 262.480 | 2.282 | |  |  |  |
|  | Cross | 1 | 0.138 | 0.138 | 8 | 999 | | 0.799 |
|  | Residuals | 115 | 258.551 | 2.248 |  |  | |  |

^z^ The clr_dist_matrix is created from the centered-log ratio on 16s sequence data.

^y^ Number of permutations

**Supplementary Table 6.** Permutational analysis of variance (PERMANOVA) and homogeneity of dispersion tests of independent variables influencing the distance matrix of centered-log transformed data, for ITS sequence analysis of rhizosphere fungi associated with the roots of four snap bean populations grown in organically managed plots at the Lewis Brown Research Farm, OR in 2020.

| **Test** | **Model** | **Effects of Terms** | **Permutations** | **Number of Permutations** |  |  |  |
| --- | --- | --- | --- | --- | --- | --- | --- |
| PERMANOVA | ITS_clr_dist_matrix^z^ ~ Breeding History + Cross | Marginal | Free | 999 |  |  |  |
|  | **Independent Variable** | **Degrees of Freedom** | **Sum of Squares** | **R^2^** | **F** | **Pr(>F)** | **Significance** |
|  | Breeding History | 1 | 432 | 0.013 | 1.530 | 0.006 | ** |
|  | Cross | 1 | 492 | 0.015 | 1.741 | 0.004 | ** |
|  | Residual | 114 | 32216 | 0.972 |  |  |  |
|  | Total | 116 | 33133 | 1.000 |  |  |  |
|  | **Independent Variable** | **Df** | **Sum Sq** | **Mean Sq** | **F** | **N.Perm^y^** | **Pr(>F)** |
| Homogeneity of Dispersion | Breeding History | 1 | 11.190 | 11.186 | 2.455 | 999 | 0.114 |
|  | Residuals | 115 | 524.080 | 4.557 |  |  |  |
|  | Cross | 1 | 1.770 | 1.774 | 0.365 | 999 | 0.566 |
|  | Residuals | 115 | 558.690 | 4.858 |  |  |  |

^z^ The clr_dist_matrix is created from the centered-log ratio on ITS sequence data.

^y^ Number of permutations


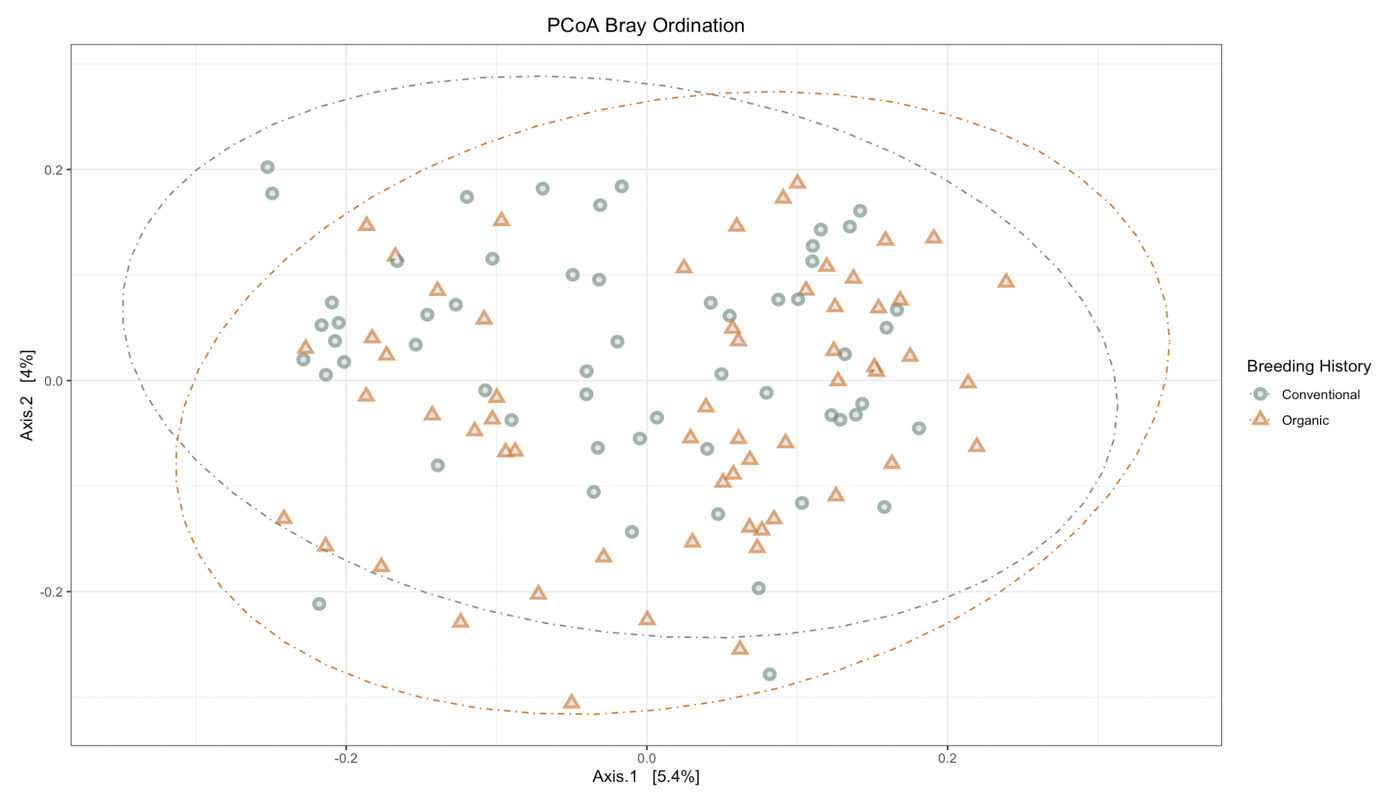


**Supplementary Figure 1.** Unconstrained ordination of breeding history in 16s data derived from rhizosphere soil samples taken from snap bean roots, according to breeding history.

**
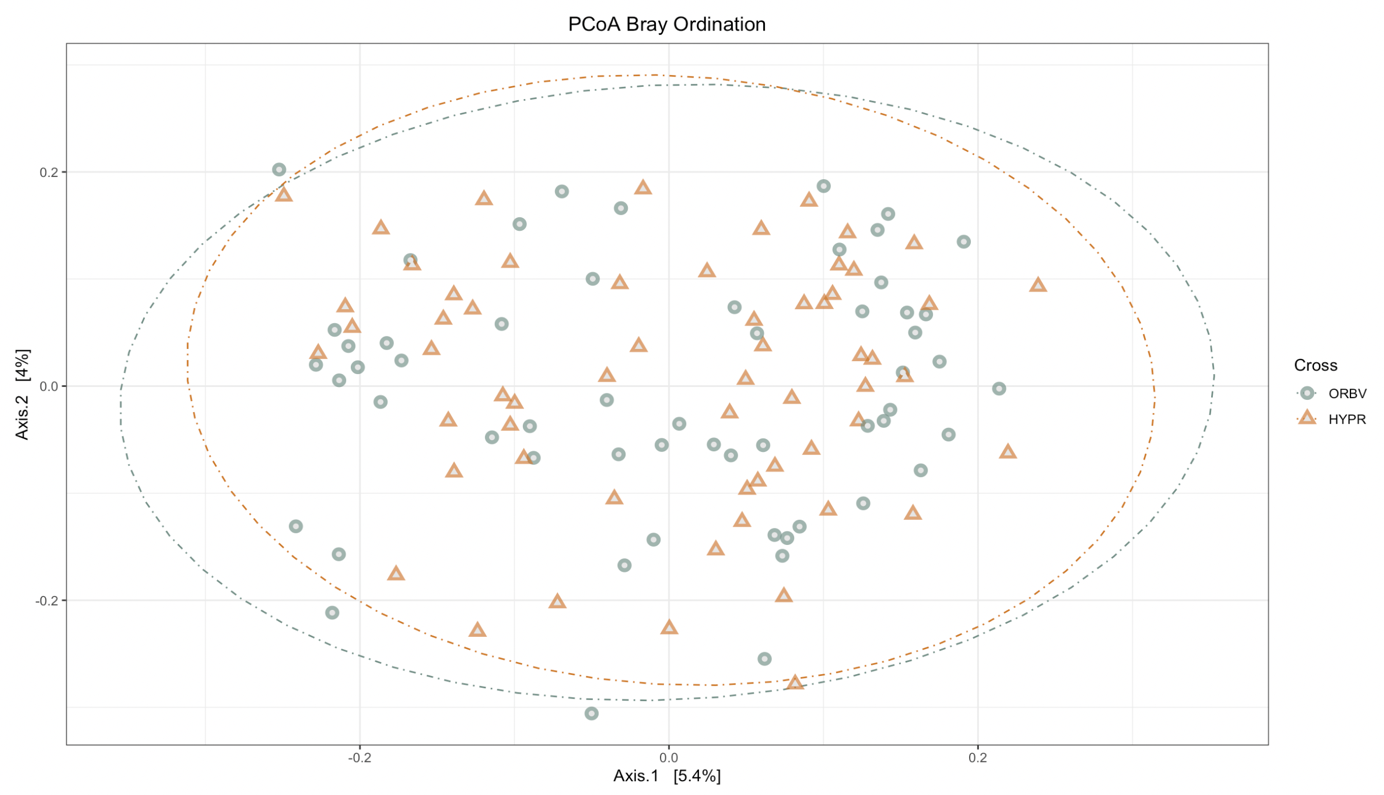
**

**Supplementary Figure 2.** Unconstrained ordination of cross in 16s data, derived from rhizosphere soil samples taken from snap bean roots, according to parentage.


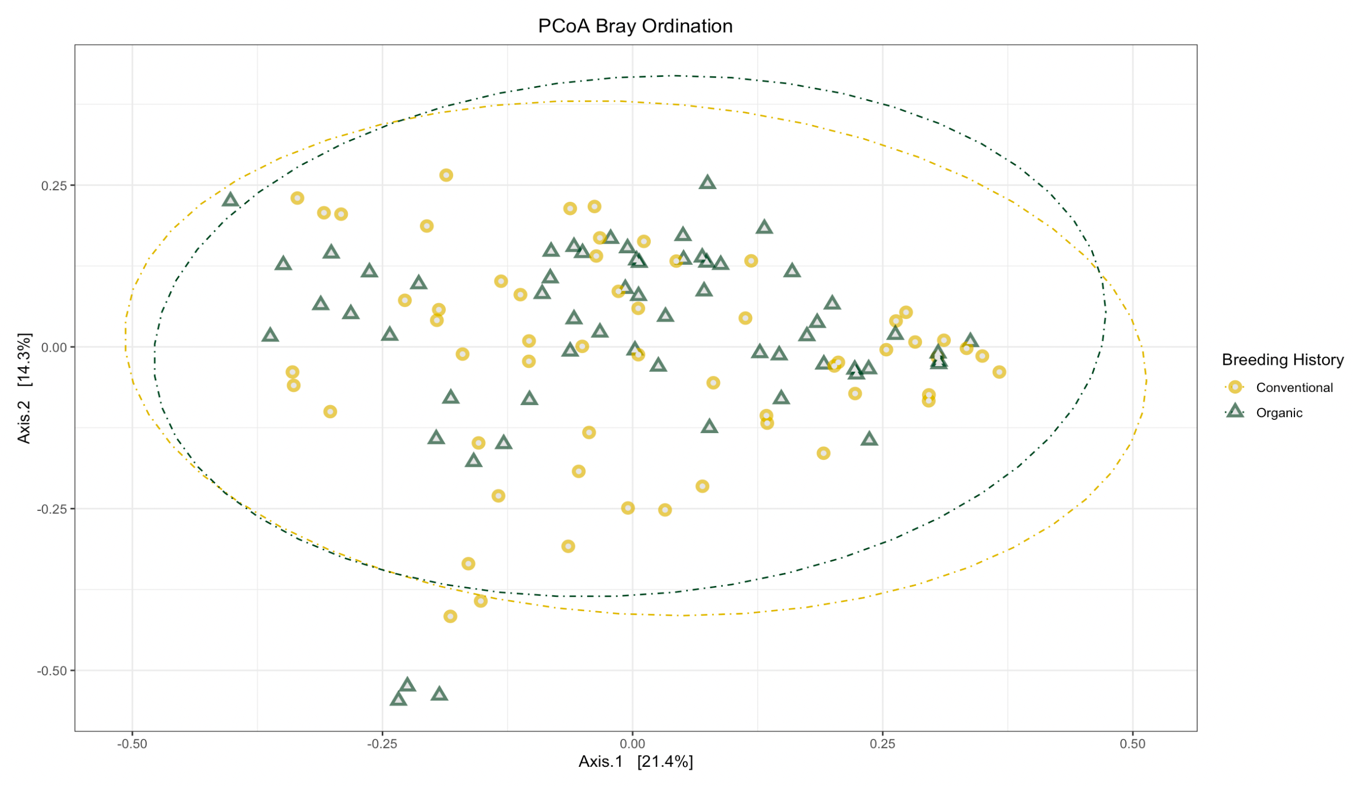


**Supplementary Figure 3.** Unconstrained ordination of breeding history in ITS data, derived from rhizosphere soil samples taken from snap bean roots, according to breeding history.


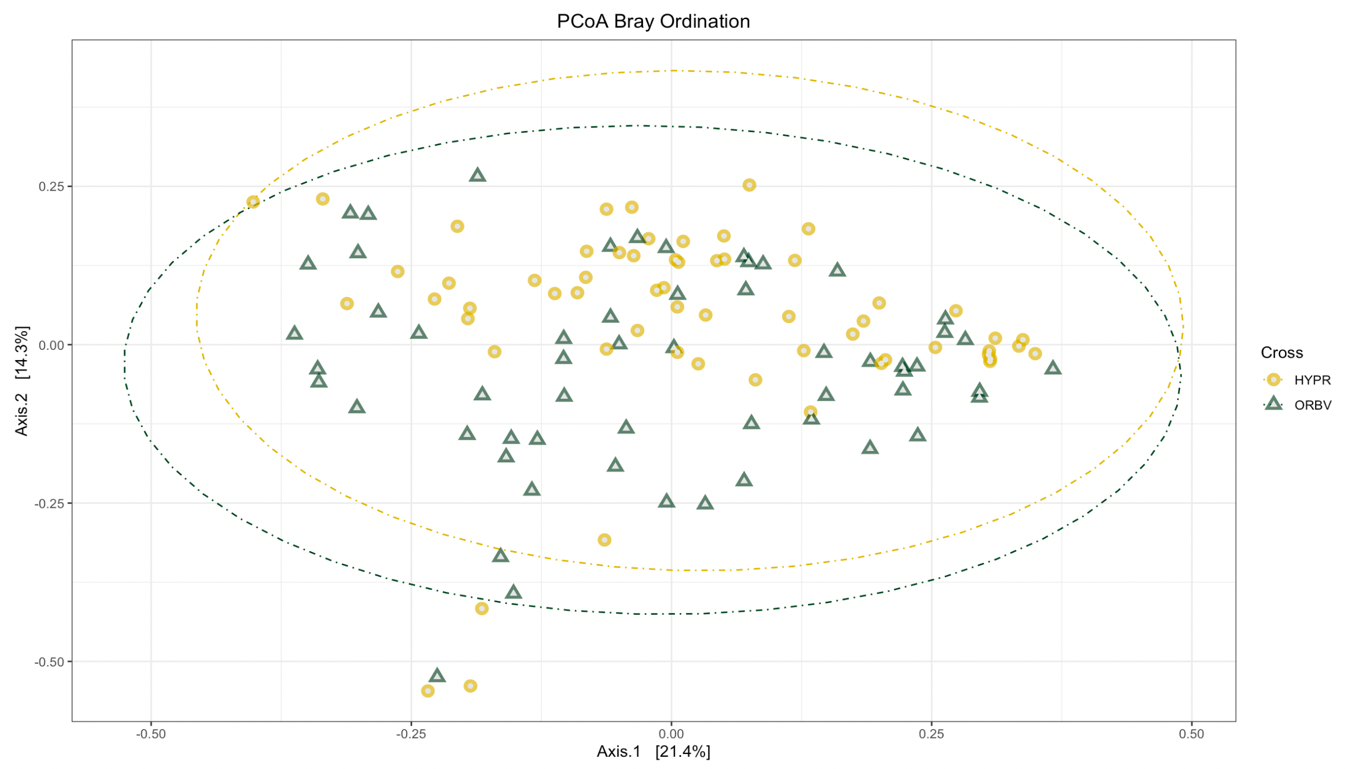


**Supplementary Figure 4.** Unconstrained ordination of cross in ITS data, derived from rhizosphere soil samples taken from snap bean roots, according to parentage.
